# Anaphylaxis as probable cause of death in a rare case of fatal *Vipera berus* bite

**DOI:** 10.1007/s12024-023-00776-2

**Published:** 2024-01-24

**Authors:** Anton F. Mittendorf, Carl Winskog

**Affiliations:** 1 Swedish National Board of Forensic Medicine, Gothenburg Unit, Box 408, 405 30 Gothenburg, Sweden; 2 Department of Oncology-Pathology, Karolinska Insitutet, Stockholm, Sweden

**Keywords:** Vipera berus, Anaphylaxis, Snake bite, Cause of death, Forensic medicine

## Abstract

This case report describes the death of a 52-year-old male who was bitten by a venomous snake, *Vipera berus* (common European adder), on his left wrist. Despite experiencing symptoms, the patient refused medical care and self-medicated with alcohol instead. He was later found dead in his residence. Autopsy and histological examination revealed evidence of an anaphylactic reaction in response to the snake bite, with additional findings of alcohol intoxication and other underlying medical conditions.

## Introduction

*Vipera berus*, the common European adder, is the only venomous snake native to Sweden [1]. Envenomation from this snake species can cause local tissue damage but rarely leads to lethal outcomes in humans [2]. The *Vipera berus* venom has shown proteolytic, fibrinolytic, anticoagulant, and phospholipase A2 activities [3]. Anaphylactic reactions, though uncommon, can occur in response to the venom [4].

## Case presentation

A 52-year-old male was found dead seated in a chair in his kitchen. According to friends, he had been bitten on the left wrist by a small snake identified as a common European adder (*Vipera berus)* the evening before his death. Despite experiencing swelling in his hand, he did not seek medical attention and instead consumed alcohol with his friends. The next morning, he was found dead.

## Clinical findings

**Fig. 1:**
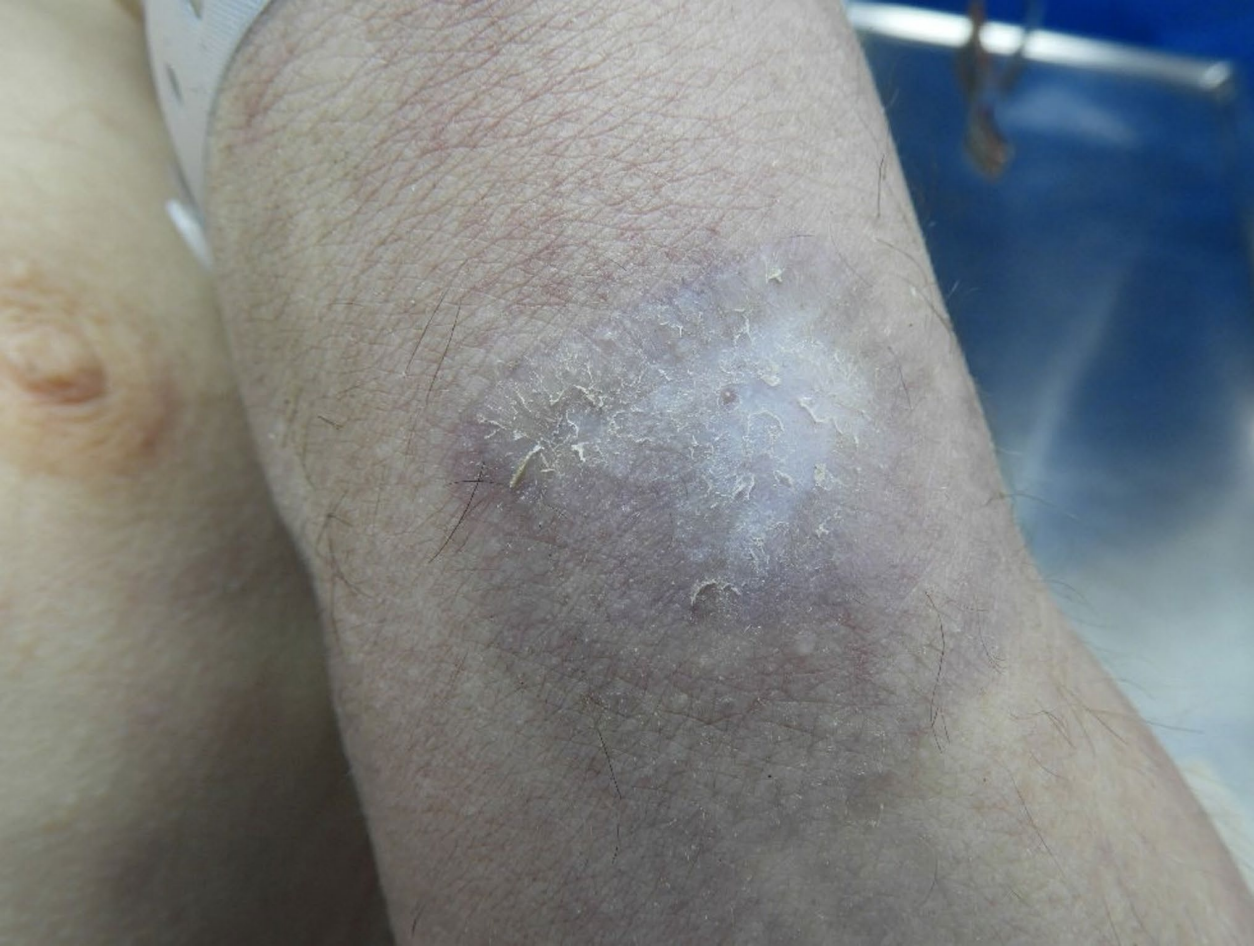
Location of bite on the left arm, just above the wrist, with a single puncture mark in a whitened patch of skin, surrounded by a greyish-brown discoloration

External examination revealed significant swelling in the left hand, along with a 2 × 2.5 cm whitened skin segment on the ulnar aspect of the left forearm, proximal to the wrist (Fig. 1, location of bite). The whitened part exhibited a single puncture mark, consistent with a snake bite, with one fang penetrating the skin. Reddish-purple discolorations were observed on various parts of the extremities, generally with irregular shapes but occasionally with a suggested annular structure. A few of these also showed partially healed excoriations, suggesting that the discolorations predated the snake bite. Therefore, the discolorations as a whole were suspected to be a pre-existing dermatological condition rather than directly linked to the cause of death.

## Autopsy findings

**Fig. 2:**
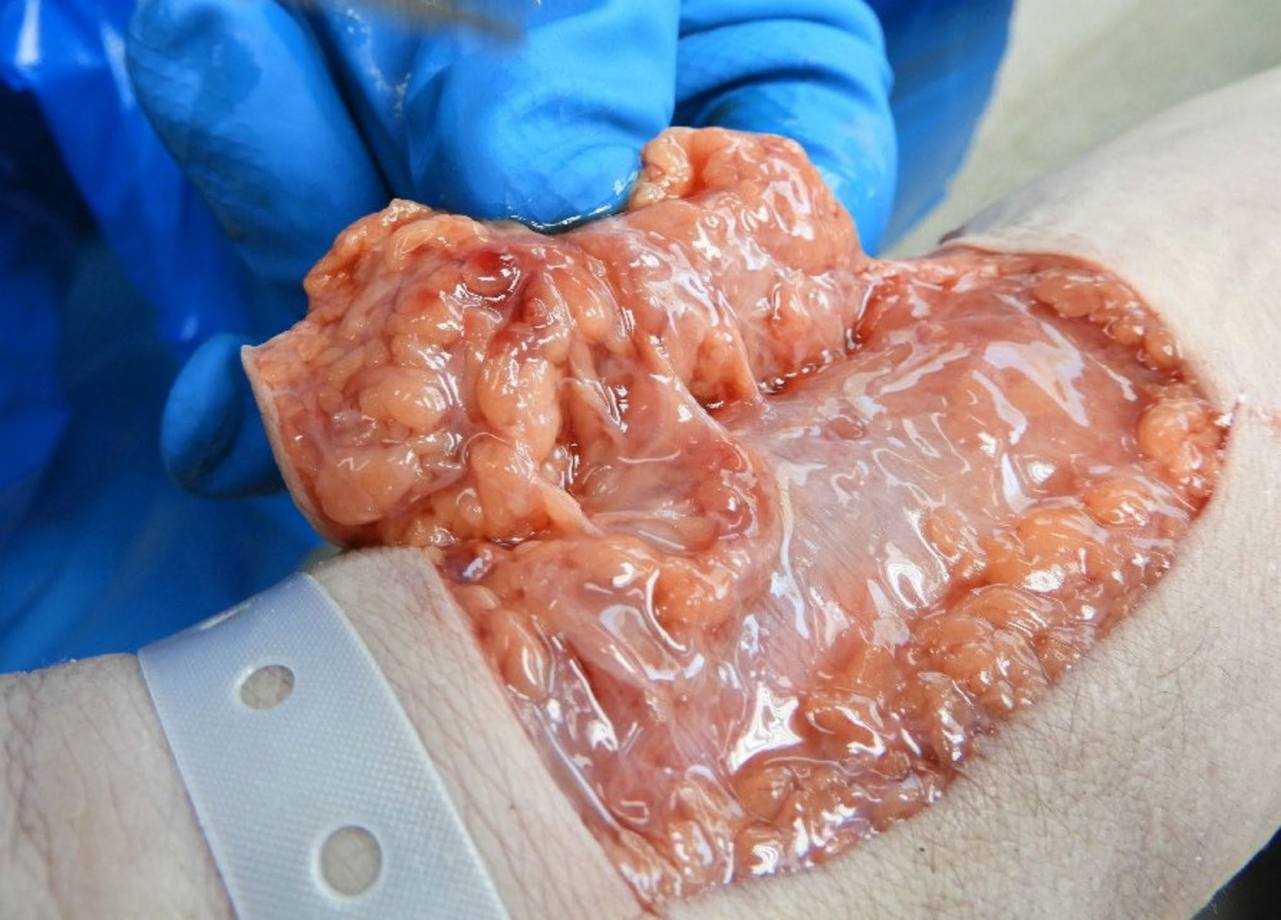
Edema in the soft tissue adjacent to the bite. In the fatty tissue of the skin flap, minimal bleeding at the puncture mark can be seen

**Fig. 3:**
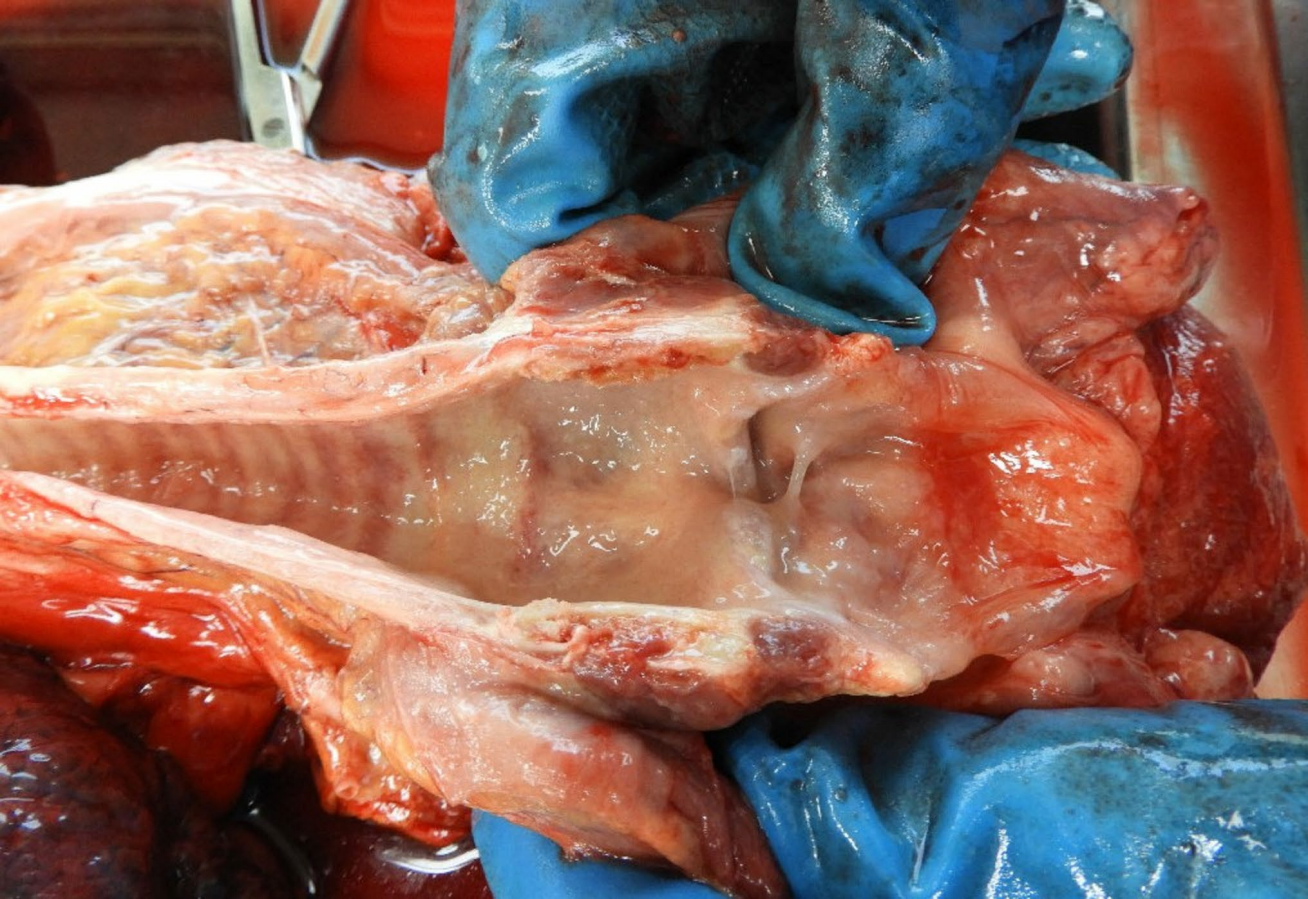
Swelling of tissue in the larynx as well as thick mucus

**Fig. 4:**
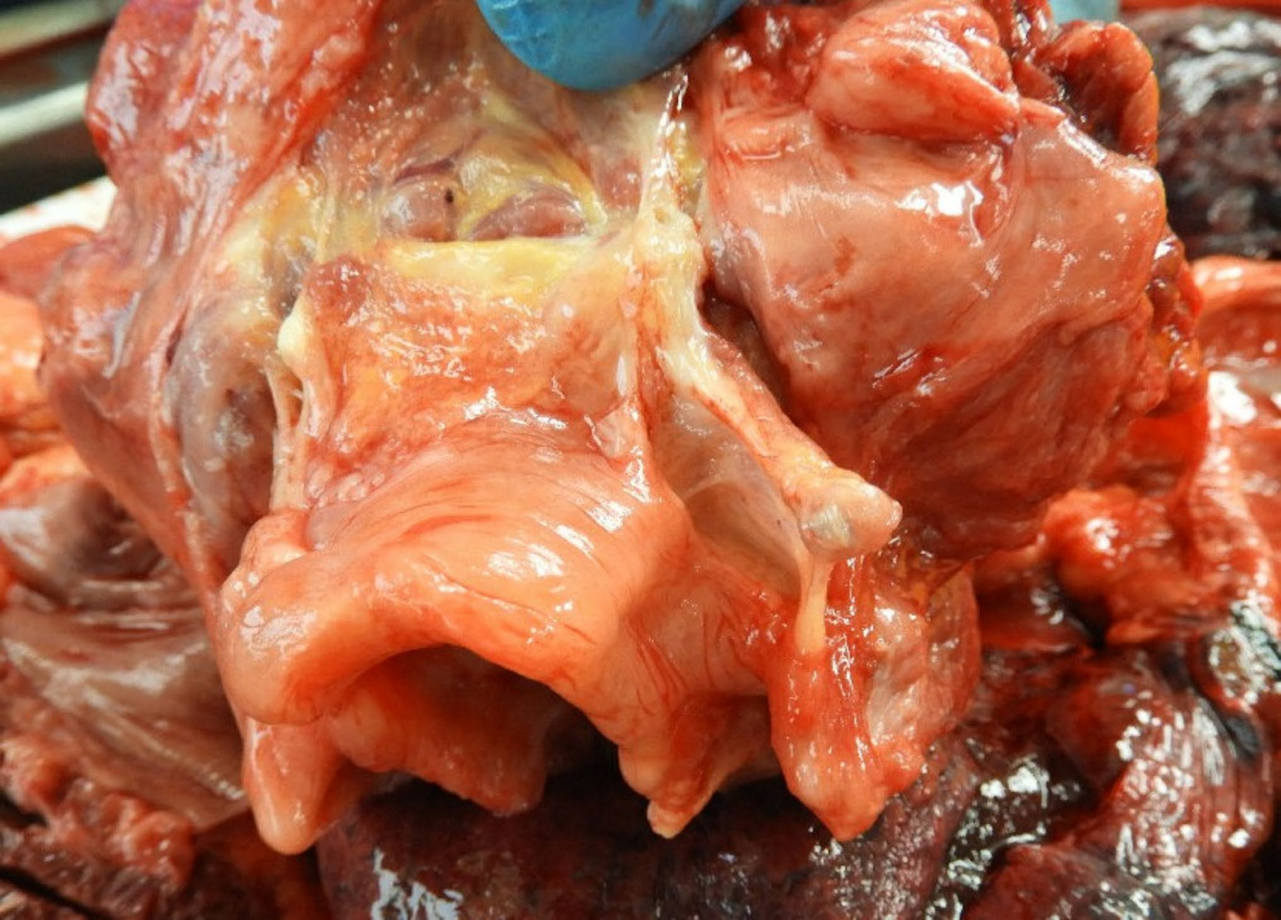
Swelling of tissue in the epiglottis, especially in the ventral aspect

During the autopsy, soft tissue adjacent to the suspected bite mark exhibited edema with continuous fluid leakage after incision (Fig. 2, edema). A thin red streak, a few millimeters in length, was observed beneath the puncture mark. Internal organs, particularly the liver and kidneys, released blood-admixed fluid during the procedure. An edematous, almost gelatinous epiglottis was seen (Figs. 3 and 4, swelling of tissue) as well as thick mucus in the larynx and trachea.

## Histological findings

Histological examinations confirmed macroscopic findings. The skin around the bite site showed a mild inflammatory reaction with occasional mast cells. The kidneys exhibited occasional glomerular thrombosis and the epiglottis showed a large number of granulocytes in the blood vessels, including eosinophils and basophils, as well as numerous tryptase-positive mast cells in the connective tissue. Additional findings without obvious direct impact on the cause of death were scar tissue in the heart and steatosis of the liver with signs of early-stage cirrhosis.

## Chemical analysis

Blood from the femoral vein and vitreous fluid revealed highly elevated ethanol content of 0.36 and 0.43% BAC, respectively, indicating alcohol intoxication. No other substances were detected. Inflammatory markers or 5HT were not examined.

## Conclusion

The cause of death was determined to be an anaphylactic reaction resulting from *Vipera berus*-envenomation. The presence of alcohol intoxication may have contributed to respiratory suppression and/or incapacitation but was deemed unlikely as the primary cause of death, as the background information indicated that the patient regularly consumed large amounts of alcohol.

This case highlights the importance of seeking prompt medical care after a venomous snake bite to prevent potentially fatal outcomes, especially in cases where anaphylactic reactions may occur. While anaphylactic reactions due to *Vipera berus*-envenomation have been described [4], the reactions seem to be rare. As there are details lacking in the background of the case, it is not possible to determine the time frame between the bite and the respiratory effects, and thus it is uncertain whether there was a delayed onset of the anaphylaxis or if the patient simply ignored the early signs, possibly due to intoxication. Further studies on the management of snake bites and associated complications in alcohol-intoxicated individuals may help improve clinical outcomes.

## Data Availability

As the National Board of Forensic Medicine's detailed personal data concerning deceased persons is subject to restictions in the Swedish Principle of Publicity, full data are not available.
